# Editorial: Treatment for Non-Small Cell Lung Cancer in Distinct Patient Populations

**DOI:** 10.3389/fonc.2022.838570

**Published:** 2022-01-27

**Authors:** Junji Uchino, Torsten Goldmann, Hideharu Kimura

**Affiliations:** ^1^ Department of Pulmonary Medicine, Kyoto Prefectural University of Medicine, Kyoto, Japan; ^2^ Bannan Central Hospital, Hamamatsu, Japan; ^3^ Pathology, Research Center Borstel, Airway Research Center North (ARCN), Member of the German Center for Lung Research (DZL), Borstel, Germany; ^4^ Department of Respiratory Medicine, Kanazawa University Graduate School of Medical Sciences, Kanazawa, Japan

**Keywords:** non-small cell lung cancer, tailored treatment, distinct patients, prognostic predictors, adverse event (AE)

Pharmacotherapy for lung cancer has changed considerably in recent years. In the 2000s, the discovery of driver genes triggered the discovery of accurate predictors of the therapeutic efficacy of molecular-targeted drugs. Additionally, recent studies have shown the effectiveness and safety of using angiogenesis suppressants and new cytotoxic anticancer drugs for distinct histological subdivisions. Subsequently, a number of driver genes were discovered, and molecular-targeted drugs were marketed. In this era, these drugs have been classified based on the genetic abnormalities they target. With the advent of immune checkpoint inhibitors against molecules such as programmed death protein 1 (PD-1) and programmed cell death ligand 1 (PD-L1), tumors are being classified according to their expression of PD-1 and PD-L1, increasing the complexities in the algorithms of drug selection and molecular testing. The results of many important clinical trials led to the establishment of various treatment modalities, which have resulted in the selection of better treatment strategies.

The development of biomarkers and novel pharmaceuticals has dramatically transformed the pharmacotherapy of lung cancer. Traditionally, the 5-year survival rate for advanced non-small cell lung cancer (NSCLC) was reported to be approximately 1–3% (1). In contrast, among driver gene-positive patients, the median survival was approximately 3 years, and the 5-year survival rate extended to 30% (1). Even in cases without driver gene mutations, the advent of immune checkpoint inhibitors has resulted in the 5-year survival rate increasing to more than 15% (2). However, these are the results of clinical trials, and actual clinical practice includes patients that differ from the standard patient population enrolled in clinical studies. There is little information available on the best treatment modalities for distinct patient populations, such as those who exhibit poor performance status, those who are elderly, or those with brain metastases, for whom the standard treatment regimens (e.g., platinum combination therapy or immune checkpoint inhibitors with chemotherapy) are deemed unsuitable.

Thus, in this Research Topic, we aimed to collect research tailored to these distinct patient populations and discuss novel studies that pinpoint special molecular subtypes of NSCLC. We were excited to receive 59 contributions, and 31 articles authored by more than 260 researchers from various countries in the fields of cancer biology, pharmacology, and therapeutics, were finally selected for inclusion in this Research Topic. Below, we have summarized the results of these studies.

## Early-Stage Lung Cancer

Wang et al. performed an analysis of driver genes using surgical specimens of combined subtypes of large-cell neuroendocrine carcinoma and SCLC respectively and reported that clinical phenotypic differences may impact the prognostic outcome in combined large-cell neuroendocrine carcinoma. Yang et al. examined the prognostic role of inflammatory biomarkers and epidermal growth factor receptor (EGFR) mutations with trimodality therapy in locally advanced lung cancer and reported that more intensive adjuvant treatment may be needed for patients with high pretreatment systemic immune-inflammation and systemic inflammation response indices, stage T2 disease, and EGFR mutations. Ji et al. reported the efficacy of computed tomography-guided stereotactic ablative brachytherapy for unresectable early-stage lung cancer and speculated that patients with stage T1 disease < 1 cm from the chest wall may have better outcomes. Lei et al. conducted a systematic review and meta-analysis of the efficacy of postoperative radiotherapy in patients with resectable stage III-N2 NSCLC and reported that it may not result in improved overall survival (OS). Hu et al. reported the usefulness of a metabolism-related gene-pair index in selecting adjuvant therapy for early-stage pulmonary adenocarcinoma. Shen et al. reported the usefulness of hypofractionated radiotherapy as an alternative therapy for patients with NSCLC not amenable to surgery or conventional chemoradiotherapy. Li et al. found that folate receptor-positive circulating tumor cell level could be a promising prognostic marker after surgery.

## Advanced-Stage Lung Cancer

Jiang et al. performed an indirect comparison of nivolumab + ipilimumab + two cycles of chemotherapy and pembrolizumab + chemotherapy for advanced NSCLC using relevant databases and reported that among women who had never smoked, a better OS could be expected with pembrolizumab + chemotherapy than with nivolumab + ipilimumab+ chemotherapy. Daniello et al., Wang et al., and Morimoto et al. studied the association between immune-related adverse events and the efficacy of immunotherapy, and Morimoto et al. reported that immune-related adverse events were associated with a better therapeutic response, particularly when immunotherapy was combined with chemotherapy.

## Gene Mutation

Takamori et al. reviewed the present treatment strategies and unresolved challenges for lung cancer with RET fusion, which is a rare mutation. Li et al. examined the cost-effectiveness of lorlatinib for anaplastic lymphoma kinase (ALK)-positive lung cancer and reported that lorlatinib was unlikely to be cost-effective compared with crizotinib for patients with previously untreated advanced ALK-positive NSCLC at a willingness-to-pay threshold of $200,000/quality-adjusted life year. Yang et al. investigated the efficacy of EGFR-tyrosine kinase inhibitor (TKI) plus chemotherapy versus EGFR-TKI monotherapy in advanced EGFR-mutant lung adenocarcinoma patients with co-mutations and reported that concurrent *TP53* mutations were found to be risk factors for EGFR-TKI monotherapy, but TKI combined with chemotherapy could eliminate this heterogeneity. Zhao et al. analyzed approximately 3000 coexisting oncogenic drivers in NSCLC and found that approximately 1.5% of NSCLC patients harbored oncogenic drivers that may coexist with EGFR mutations. Choi et al. discussed the potential of artificial intelligence-based screening and drug discovery in the development of novel drugs for acquired resistance to EGFR mutations. Feng et al. examined the efficacy and safety of EGFR-TKI combined with thymosin for advanced NSCLC patients with active EGFR mutation, and they reported that the combination therapy significantly prolonged progression-free survival and OS compared with EGFR-TKI monotherapy without increasing adverse events. Song et al. reported that administration of afatinib resulted in promising outcomes for the NSCLC patients with *HER2* mutations and amplification.

## Others

Hou et al. performed a systematic review of locally advanced hepatoid adenocarcinoma of the lung with PIK3CA mutations and concluded that only radical surgery can significantly improve outcomes. Xu et al. discussed the tumor microenvironment and concluded that angiotensin-converting enzyme 2/angiotensin-converting enzyme inhibitors promote vasculogenic mimicry formation *via* Nodal/Notch4 activation and lead to a strong and solid structure of vasculogenic mimicry *via* inhibition of vascular endothelial-cadherin internalization. Li et al. focused on developing a single-needle cone puncture technique in Iodine-125 seeds brachytherapy for people with advanced thoracic malignancies and reported its efficacy. Wu et al. reported the usefulness of ivosidenib, an anti-leukemia drug, in lung cancer, using transcriptomic analysis.

In conclusion, as the study of gene mutations and biomarkers progresses in the future, individualization of treatment strategies will increase, and formulation of safe and effective treatment for older patients will benefit the growing aging population.

As a result, we are convinced that the content of the papers included in this Research Topic will be extremely valuable in guiding further research.

## Author Contributions

JU, TG, and HK wrote and reviewed this editorial. All authors contributed to the article and approved the submitted version.

## Conflict of Interest

The authors declare that the research was conducted in the absence of any commercial or financial relationships that could be construed as a potential conflict of interest.

## Publisher’s Note

All claims expressed in this article are solely those of the authors and do not necessarily represent those of their affiliated organizations, or those of the publisher, the editors and the reviewers. Any product that may be evaluated in this article, or claim that may be made by its manufacturer, is not guaranteed or endorsed by the publisher.
